# A Practical Weakly Supervised Framework for Dose-Up Translation of Low-Enhanced CT Under Clinical Acquisition Variability

**DOI:** 10.3390/jimaging12050190

**Published:** 2026-04-27

**Authors:** Jong Bub Lee, Se Hwan Lim, Yu Jin Jung, Jae Hwan Kim, Hyun Gyu Lee

**Affiliations:** 1 Department of Electrical and Computer Engineering, Inha University, 100 Inha-ro, Incheon 22212, Republic of Korea; bub3690@inha.edu; 2 Department of Electronic Engineering, Inha University, 100 Inha-ro, Incheon 22212, Republic of Korea; tpghks726@inha.edu; 3 Department of Artificial Intelligence Semiconductor Engineering, Inha University, 100 Inha-ro, Incheon 22212, Republic of Korea; 4 Department of Veterinary Medical Imaging, College of Veterinary Medicine, Konkuk University, 120 Neungdong-ro, Seoul 05029, Republic of Korea; yujin970430@gmail.com; 5 College of Medicine, Inha University, 100 Inha-ro, Incheon 22212, Republic of Korea

**Keywords:** low-dose contrast-enhanced CT, weak spatial alignment, veterinary abdominal CT, structure-preserving enhancement, image translation

## Abstract

Low-dose contrast-enhanced computed tomography (CT) is widely used to reduce contrast-induced toxicity, but reduced iodine concentration and inconsistent acquisition conditions often produce uneven contrast attenuation and spatial misalignment between scans. In this context, we define dose-up translation as the computational process of synthetically enhancing low-dose contrast images to approximate the visual and diagnostic quality of full-dose acquisitions. These factors limit the effective use of routinely acquired imaging data for dose-up translation, particularly in veterinary abdominal CT where respiratory motion and postural variability further degrade anatomical correspondence. We present a weakly aligned enhancement framework designed to operate under spatial misalignment and limited paired data. Registration-based pseudo-references are constructed using a hybrid strategy that combines deformable anatomical alignment with feature-level correspondence. Dose-up translation is performed using structure-preserving translation with multi-scale consistency and edge-aware regularization to maintain anatomical boundaries. To address limited low-dose datasets, a two-stage knowledge transfer strategy transfers anatomical and contrast priors from abundant pre-contrast data. Quantitative evaluation demonstrated region-level contrast-to-noise ratio improvements of up to 31.5% (e.g., from 5.55 to 8.38 in the caudal vena cava (CVC), *p* < 0.05) compared with baseline enhancement methods across 1171 test slices. Experiments demonstrate consistent improvements in structural fidelity, distributional realism, and region-level vascular conspicuity compared with paired, unpaired, and synthetic-pairing baselines. These findings suggest that the dose-up translation of low-enhanced CT is better formulated as a weakly aligned domain adaptation problem rather than a strictly paired reconstruction task, enabling practical image translation under realistic clinical acquisition variability.

## 1. Introduction

Contrast-enhanced computed tomography (CT) plays a central role in diagnostic imaging by improving the visualization of vascular structures and soft tissues. In routine clinical environments, however, contrast-enhanced acquisitions are frequently constrained by safety considerations, heterogeneous imaging protocols, and inconsistent patient conditions. These factors often result in reduced contrast conspicuity and spatial inconsistency between examinations, limiting the effectiveness of image enhancement techniques when applied to routinely acquired clinical data.

In practice, repeated CT scans rarely maintain strict anatomical correspondence. Respiratory motion, postural variability, and subject-specific anatomical differences introduce spatial misalignment that challenges conventional enhancement approaches relying on precise voxel-wise matching. When geometric alignment is weak, pixel-level supervision may produce structural distortions, whereas fully unpaired translation methods often fail to preserve clinically meaningful contrast patterns, particularly in vascular structures. As a result, large volumes of retrospectively collected imaging data remain underutilized for enhancement tasks due to acquisition variability and inconsistent correspondence [1,2].

These challenges are particularly pronounced in veterinary imaging, where iodinated contrast media must be administered cautiously to reduce adverse effects including contrast-induced nephropathy, thyroid dysfunction, and allergic reactions [3]. The growing prevalence of chronic kidney disease and aging animal populations further motivates the adoption of reduced-dose contrast protocols [4,5]. Although low-dose contrast-enhanced CT improves patient safety, reduced iodine concentration produces sparse and spatially uneven contrast attenuation patterns that complicate image interpretation. Importantly, these patterns do not represent patchy intra-organ heterogeneity but rather structure-dependent attenuation differences across tissues under reduced iodine dose.

In canine abdominal CT, recommended iodine doses range from 600 to 880 mgI per kilogram of body weight, yet substantial inter-individual variability complicates dose optimization [6,7]. Consequently, low-dose acquisitions (Post40) retain partial enhancement signals but lack reliable spatial correspondence with full-dose scans (Post100). This setting differs fundamentally from conventional non-contrast-to-contrast synthesis, as enhancement information is incomplete rather than absent. Effective enhancement under these conditions therefore requires strategies capable of operating without strict spatial alignment.

Previous CT enhancement studies have primarily focused on synthesizing full-contrast images from non-contrast scans [8,9]. Such approaches generally assume stable spatial correspondence and often emphasize downstream segmentation or reconstruction tasks rather than direct enhancement of diagnostic image quality. GAN-based methods including pix2pix have demonstrated vulnerability to breathing- and position-induced misalignment [10,11], a limitation that becomes more severe in veterinary imaging due to interspecies anatomical variability and limited motion control.

**Figure 1:**
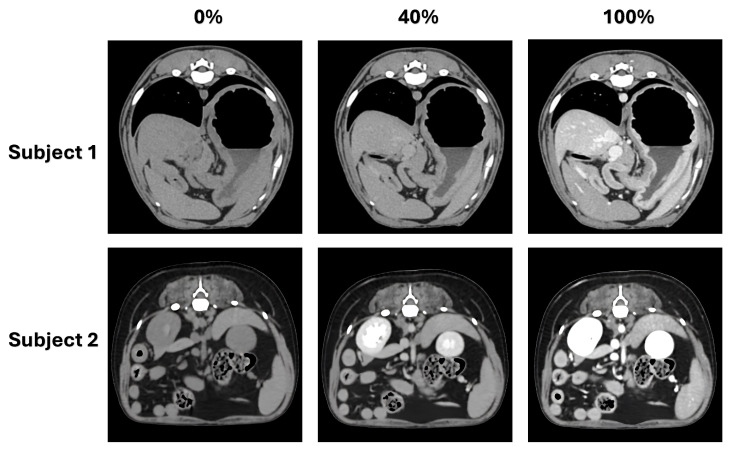
Comparison of abdominal CT images across contrast dose levels. For each subject, non-contrast (0%), low-dose contrast-enhanced (Post40), and full-dose contrast-enhanced (Post100) images are shown. Full-dose images provide clearer delineation of vascular and parenchymal structures, whereas low-dose images exhibit reduced and spatially uneven contrast attenuation. Mild spatial discrepancies between acquisitions are also observable, reflecting realistic variations caused by respiratory motion and postural differences.

Figure 1 illustrates contrast variations across non-contrast, low-dose, and full-dose CT acquisitions. Full-dose images provide clear delineation of vascular structures and parenchymal organs, whereas low-dose images exhibit sparse and spatially inconsistent enhancement. Respiratory motion and anatomical variability further exacerbate misalignment, rendering direct application of conventional synthesis models unreliable. These observations highlight the need for enhancement strategies that tolerate spatial inconsistency while preserving anatomical structure.

Consequently, dose-up translation of low-enhanced scans under routine clinical acquisition should not be treated as a strictly paired reconstruction task. In this context, we define weakly aligned domain adaptation as a learning paradigm that translates images between distinct clinical domains (e.g., from low-enhanced to full-dose appearances) by leveraging global anatomical priors and feature-level similarities, without relying on strict, pixel-perfect spatial correspondence. Instead, it represents a practical enhancement problem constrained by spatial misalignment and heterogeneous imaging conditions, requiring learning strategies that remain robust without precise voxel-level correspondence.

To address this challenge, we present a weakly aligned enhancement framework designed for CT imaging scenarios where precise spatial correspondence is unavailable. The proposed approach leverages registration-based reference construction and progressive knowledge transfer to enable structure-preserving enhancement under realistic acquisition variability.

The main contributions of this work are summarized as follows:

1. **Imaging problem reformulation under weak alignment:** We formulate the dose-up translation of low-enhanced CT as an imaging task performed under imperfect spatial correspondence rather than as a strictly aligned reconstruction problem.
2. **Weakly aligned enhancement framework:** We introduce a hybrid registration and enhancement pipeline that enables structure-preserving contrast improvement using imperfectly aligned data.
3. **Practical validation in veterinary CT:** We demonstrate anatomically consistent dose-up translation in veterinary abdominal CT, improving vascular and organ conspicuity while maintaining structural boundaries under realistic clinical acquisition conditions.

## 2. Related Works

**Reconstruction under Strict Correspondence:** Many medical image enhancement studies have been developed under acquisition settings where precise anatomical correspondence can be ensured. Such approaches are widely applied to accelerated MRI, low-dose CT denoising, and PET reconstruction tasks, where imaging conditions allow consistent voxel-wise alignment and physically modeled signal degradation [12,13,14,15,16]. Under these controlled scenarios, enhancement can be formulated as a reconstruction problem with direct pixel-level supervision.

However, contrast-enhanced CT acquired in routine clinical environments does not satisfy these conditions. Iodine contrast dynamics follow nonlinear pharmacokinetic processes that lack closed-form forward models, and repeated acquisitions often exhibit spatial misalignment due to patient motion and variable positioning. These characteristics limit the applicability of strictly supervised reconstruction strategies.

**Enhancement via Synthetic Pairing:** To mitigate the scarcity of paired training data, several studies have generated synthetic correspondences using physics-based simulation. For example, iodine maps derived from dual-energy CT have been used to synthesize reduced-contrast images for supervised learning [17]. While such methods enable controlled training, their reliance on simulated conditions raises concerns regarding generalizability to heterogeneous clinical acquisitions.
**Domain Adaptation in Medical Imaging:** In recent years, bridging the visual and feature gap between distinct acquisition protocols (e.g., low-dose vs. full-dose CT) has frequently been formulated as an Unsupervised Domain Adaptation (UDA) problem. Recent literature has extensively explored UDA techniques, often leveraging adversarial feature alignment or contrastive learning to map source and target distributions without strictly paired data [18,19]. For instance, state-of-the-art frameworks have integrated soft-labeled contrastive learning to improve domain alignment [18] or combined cycle consistency with hybrid contrastive learning (e.g., CycleH-CUT) to enhance unsupervised medical image translation [19]. However, standard UDA methods fundamentally prioritize global distribution matching over local spatial consistency. Consequently, when applied to weakly aligned clinical datasets characterized by heterogeneous contrast patterns, purely distribution-driven adaptation often struggles to preserve localized anatomical structures. As discussed in the subsequent approaches, whether implemented via translation networks or generative priors, this lack of explicit spatial correspondence remains a critical bottleneck.
**Translation under Limited Correspondence:** Unpaired or weakly paired translation approaches have been explored to relax strict alignment requirements. Cycle-consistent translation frameworks have been applied to contrast synthesis between non-contrast and enhanced CT domains using synthetic or loosely matched data [20,21]. Furthermore, recent advancements in unsupervised medical image processing have demonstrated the potential of transformer-based architectures for enhancement and denoising tasks without paired data [22]. Although these approaches reduce the dependency on paired datasets, their performance is strongly influenced by the realism of synthetic data and often prioritizes global appearance consistency over the preservation of fine anatomical structures.
**Joint Registration and Enhancement Strategies:** To compensate for spatial inconsistency, several studies have incorporated deformable registration into image enhancement pipelines. Joint optimization of alignment and synthesis has demonstrated improved correspondence across multiphase abdominal CT acquisitions [23]. Nevertheless, these methods remain sensitive to registration inaccuracies and typically assume consistent intensity relationships between phases. Such assumptions become unreliable in low-dose contrast-enhanced CT, where enhancement signals are spatially heterogeneous and partially preserved.
**Generative Models under Alignment Constraints:** Recent generative diffusion models have achieved high-fidelity image synthesis when accurate conditioning and large paired datasets are available. However, these approaches depend on stable voxel-wise correspondence during training and inference. In scenarios involving respiratory motion and variable acquisition conditions, misalignment can degrade conditioning reliability and introduce structural artifacts, limiting practical deployment in clinical enhancement tasks [24,25].

Overall, existing approaches primarily target imaging scenarios with controlled acquisition conditions or synthetic correspondence. In contrast, low-dose contrast-enhanced CT acquired in routine practice exhibits spatial misalignment and incomplete enhancement signals, making conventional reconstruction and translation paradigms less suitable. These limitations motivate enhancement strategies designed to operate under weak spatial alignment while preserving anatomical structure.

## 3. Proposed Framework

### 3.1. Overview

**Figure 2:**
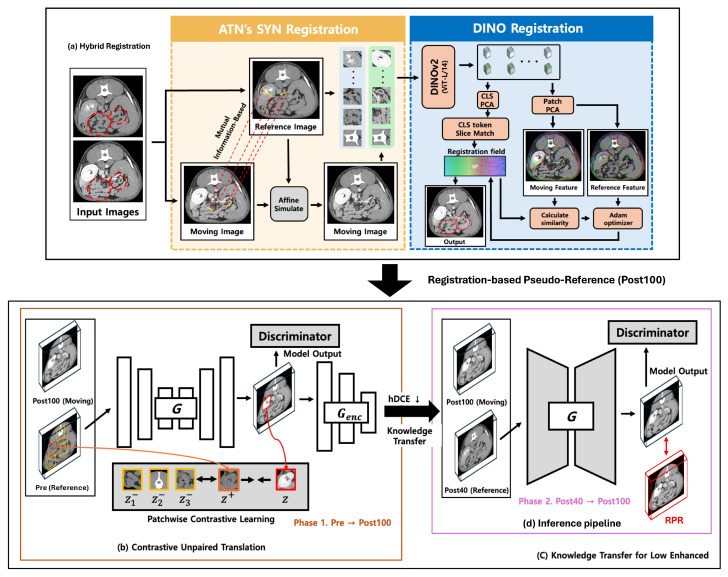
Overview of the proposed weakly aligned dose-up translation framework designed for CT acquired under imperfect spatial correspondence. The pipeline addresses the absence of reliable voxel-level pairing by progressively constructing stable supervisory signals and learning contrast-aware representations. (**a**) Hybrid registration module that generates registration-based pseudo-references (RPRs) through sequential global deformable alignment (ANTs-SyN) followed by feature-level refinement (DINO-Reg), improving anatomical correspondence under heterogeneous contrast distributions. (**b**) Structure-preserving contrastive translation stage that enhances contrast while maintaining anatomical boundaries under weak alignment by leveraging patchwise contrast-aware representation learning. (**c**) Two-stage knowledge transfer strategy that first learns general contrast characteristics from full-dose data and subsequently adapts to low-dose acquisitions, enabling stable optimization with limited and imperfect datasets. (**d**) Inference pipeline enabling direct enhancement of clinical low-dose CT without requiring paired reference images, supporting practical deployment under routine acquisition variability.

This study presents a weakly aligned enhancement framework designed for contrast-enhanced CT acquired under spatial misalignment and limited paired data. The overall processing pipeline is illustrated in Figure 2.

Rather than relying on precisely aligned image pairs, the framework is structured to operate under realistic acquisition conditions where anatomical correspondence between scans is imperfect. The proposed pipeline integrates registration-based reference construction, structure-preserving enhancement, and progressive knowledge transfer to enable robust contrast improvement from routinely acquired clinical data.

The framework operates in two sequential stages. First, enhancement behavior is learned from abundantly available full-contrast examinations to establish general anatomical and contrast relationships. Second, the learned enhancement patterns are adapted to low-dose acquisitions, enabling reliable contrast improvement despite limited training data and incomplete enhancement signals.

Two functional modules support this process. A hybrid registration module constructs reference-aligned image pairs under weak spatial correspondence, and a structure-preserving enhancement module improves contrast while maintaining anatomical boundaries without requiring pixel-level annotations.

### 3.2. Hybrid Registration for Weak Alignment

To compensate for spatial inconsistency between acquisitions, a two-stage registration strategy is employed (Figure 2a). Fixed images (Pre or Post40) are aligned with moving images (Post100) to construct reference-consistent pairs.

The first stage performs global anatomical alignment using deformable registration based on mutual information optimization. This step establishes coarse correspondence across major anatomical structures.

The second stage refines local alignment using feature-level correspondence that is robust to intensity variation. In low-dose CT images, sparse enhancement patterns can destabilize intensity-based registration; therefore, contrast-invariant feature matching is employed to improve patch-level alignment reliability.

The resulting weakly aligned image pairs are converted into registration-based pseudo-references (RPRs), which serve as supervisory anchors for subsequent enhancement while avoiding the need for strict voxel-wise correspondence.

To ensure reproducibility, the first stage achieved global alignment using the Symmetric Normalization (SyN) transformation [26,27], optimized over a multi-resolution pyramid with 100, 70, and 20 iterations. The second stage refined local features using a pre-trained DINOv2 model, specifically the ViT-L/14 architecture with registers. These registers mitigate common artifacts in standard ViT models, yielding robust, contrast-agnostic representations. Feature extraction utilized a patch size of 14 and an embedding dimension of 1024. Finally, feature matching was optimized via the ConvexAdam algorithm for 1000 iterations (learning rate 3.0, smooth weight 2.0), applying a lung area mask to constrain the registration field [28,29].

### 3.3. Structure-Preserving Dose-Up Translation

Dose-up translation is performed using a weakly supervised translation module designed to operate with imperfectly aligned references. Rather than enforcing pixel-wise reconstruction, enhancement is guided by correspondence-aware contrast learning and structural regularization to preserve anatomical consistency.

The enhancement module improves contrast while maintaining vessel and organ boundaries through two complementary mechanisms: (i) contrast-aware representation matching between input and reference images and (ii) multi-scale structural constraints that stabilize enhancement under spatial inconsistency.

To prevent artificial texture generation and over-smoothing, regularization terms are introduced to preserve anatomical edges and global structural coherence. These constraints enable contrast improvement while minimizing distortion of clinically relevant structures.

#### 3.3.1. Structure-Aware Regularization

To maintain anatomical consistency during enhancement, multi-scale and edge-preserving regularization are incorporated.

The multi-scale pyramid constraint is defined as

(1) $$L_{\mathrm{pyr}}=\sum_{s}\lambda_{s}L_{1}\left(G\left(x_{s}\right),y_{s}\right),$$

which enforces agreement across spatial resolutions, improving global anatomical stability under imperfect correspondence, where $x_{s}$ represents the input image at spatial scale *s*, $G(x_{s})$ is the generated image, $y_{s}$ is the registration-based pseudo-reference (RPR), and $\lambda_{s}$ is the scale-specific weight.

The edge-preserving constraint is defined as

(2) $$L_{\mathrm{edge}}={∥\nabla G(x)-\nabla y∥}_{1}+{∥\nabla^{2}G\left(x\right)-\nabla^{2}y∥}_{1}$$

which enforces first- and second-order gradient consistency to preserve vessel and organ boundary sharpness, where G(x) denotes the generated image, *y* is the registration-based pseudo-reference (RPR), and ∇ and ∇^2^ represent the first-order gradient and second-order Laplacian operators, respectively.

The combination of PatchNCE and $L_{\mathrm{edge}}$ acts as a robust regularizer against noise amplification. PatchNCE promotes learning of shared semantic features at the patch level rather than pixel-level intensities, while the gradient consistency imposed by $L_{\mathrm{edge}}$ penalizes high-frequency stochastic variations, suppressing artificial noise commonly observed in standard GAN-based translations. Together, these constraints ensure that dose-up translation remains anatomically plausible under weak alignment.

#### 3.3.2. Comprehensive Loss Function

The final objective for our weakly supervised enhancement framework combines the adversarial loss, structure-aware regularizations (multi-scale pyramid $L_{1}$ and edge-preserving losses), and contrastive learning components (hard-negative decoupled contrastive and semantic-relation consistency losses). The total loss optimized by the generator is defined as

(3) $$\begin{array}{cc} L_{\mathrm{total}}\;= & \;\lambda_{\mathrm{adv}}L_{\mathrm{adv}}+\lambda_{\mathrm{SRC}}L_{\mathrm{SRC}}+\lambda_{\mathrm{HDCE}}L_{\mathrm{HDCE}}\\ \; & +\lambda_{\mathrm{pyr}}L_{\mathrm{pyr}}+\lambda_{\mathrm{edge}}L_{\mathrm{edge}} \end{array}$$

where $\lambda_{\mathrm{adv}},\;\lambda_{\mathrm{SRC}},\;\lambda_{\mathrm{HDCE}},\;\lambda_{\mathrm{pyr}}$, and $\lambda_{\mathrm{edge}}$ denote the weighting coefficients for the adversarial, semantic-relation consistency, hard-negative decoupled contrastive, multi-scale pyramid $L_{1}$, and edge-preserving loss terms, respectively. The specific values of these hyperparameters are provided in Section 4.1.3.

### 3.4. Knowledge Transfer Strategy

To address the limited availability of low-dose contrast data, a two-stage knowledge transfer strategy is employed (Figure 2c).

In the first stage, enhancement characteristics are learned from large-scale full-contrast examinations, enabling the model to capture general anatomical and contrast relationships. In the second stage, the learned representations are adapted to low-dose acquisitions, allowing reliable enhancement despite reduced contrast signals and limited training samples.

This staged adaptation stabilizes enhancement performance under realistic clinical constraints where paired data are scarce and spatial correspondence is imperfect.

## 4. Experimental Evaluation

### 4.1. Dataset and Imaging Protocols

#### 4.1.1. Dataset

All CT images were acquired at the College of Veterinary Medicine, Konkuk University, using a Canon Aquilion Lightning scanner (160-channel detector). This study was approved by the Institutional Animal Care and Use Committee (IACUC) of Konkuk University (Approval No. KU 24109, approved on 11 June 2024).

The imaging protocol used 120 kV tube voltage and 110–135 mAs tube current–time product, with a trigger interval of 750 ms. The contrast-enhanced CT scans were acquired using iohexol (350 mg iodine/mL; Omnipaque, GE Healthcare). The contrast medium was administered at an injection rate of 2 mL/s with saline dilution. To ensure a consistent venous phase, post-contrast images were acquired 60 s after contrast injection.

The spatially uneven contrast attenuation observed in low-dose scans (Post40) is primarily attributed to reduced total iodine dose combined with inter-individual pharmacokinetic variations among veterinary subjects, rather than suboptimal bolus timing.

The dataset comprised CT scans from 168 subjects in total, with non-overlapping cohorts for pre-training (Phase 1), fine-tuning (Phase 2), and evaluation. For the first stage of knowledge transfer, Pre–Post100 image pairs from 156 subjects were used. The second stage used Post40–Post100 data from 8 subjects for low-dose adaptation. An independent evaluation cohort consisted of 4 subjects. This split was designed to prevent subject-level information leakage across training, adaptation, and evaluation.

To mitigate overfitting on the limited low-enhanced cohort (n=8), we employed a two-stage transfer learning strategy. The model was pre-trained (Phase 1) on a larger dataset (n=156) of pre-contrast and full-dose image pairs to establish robust anatomical priors. For subjects who underwent all three acquisitions (Pre, Post40, and Post100, such as those illustrated in Figure 1), they were exclusively assigned to either the Phase 2 or evaluation cohorts. Their corresponding pre-contrast images were strictly excluded from the Phase 1 dataset to ensure complete, non-overlapping patient separation and prevent data leakage.

Furthermore, consecutive scans were acquired within a single clinical session. The institutional protocol dictated a strict 10-minute interval between scans to allow for sufficient contrast clearance and minimize cumulative dosing effects; consequently, the median interval was 10 min, with a maximum interval of 12 min due to minor clinical workflow variations. While this regulated interval was necessary for patient safety, it inevitably introduced non-linear organ deformations due to respiratory motion and postural shifts over time. This inherent physiological variability makes strict voxel-wise correspondence practically impossible, further justifying our weakly aligned learning framework.

To provide weak supervisory anchors, registration-based pseudo-reference (RPR) images were constructed. In the first stage, Post100 images were registered as moving images to Pre images used as fixed references. In the second stage, Post100 images were registered as moving images to Post40 images used as fixed references.

#### 4.1.2. Preprocessing

All CT volumes were preprocessed using a standardized pipeline prior to training and evaluation. Intensity values were clipped to a soft-tissue Hounsfield unit (HU) window (center 40, width 400) and normalized to [0, 1]. For evaluation and visualization, normalized images were converted to 8-bit format ([0, 255]), whereas all network training was performed on normalized floating-point data.

All models were trained slice-wise in 2D. Although the evaluation cohort was limited, slice-wise analysis enabled assessment across diverse anatomical cross-sections. Volumetric consistency was examined separately in post hoc analyses.

Images were resampled to a uniform voxel spacing of 0.5 × 0.5 × 2.0 mm using linear interpolation, and orientation matrices were aligned to ensure consistent anatomical direction across subjects. Resampled volumes were rotated to a common reference orientation, and each axial slice was cropped or zero-padded to 512 × 512 pixels. Due to GPU memory constraints, all images were resized to 256 × 256 pixels for training.

It is important to note that no manual inspection, specific anatomical landmarking, or subjective cropping was involved in defining the initial volumes or extracting specific regions (e.g., the abdominal or kidney levels). Instead, the entire acquired volume from the scanner was utilized for each phase (Pre, Post40, and Post100). To establish correspondence automatically, all volumes were first standardized to a uniform physical scale by resampling them to a 2.0 mm spacing along the z-axis using linear interpolation.

Following this standardization, deterministic inter-slice pairing was established by matching the absolute physical z-coordinates to identify the nearest neighbor pairs. During this process, any surplus slices at the cranial or caudal ends that lacked a corresponding pair within the shared physical range were automatically excluded. Consequently, the overlapping spatial regions were naturally defined as a byproduct of this mathematical intersection, ensuring highly reproducible slice-level correspondence without relying on subjective visual assessments of global landmarks.

While this approach provides consistent slice-level correspondence, residual anatomical misalignment across scans inevitably remains due to physiological motion. This residual misalignment is subsequently addressed by the hybrid registration module. After this initial preprocessing and slice pairing, DINO-Reg was applied to construct registration-based pseudo-reference (RPR) images for subsequent enhancement learning.

Importantly, our framework does not assume or enforce exact anatomical-level correspondence (e.g., organ-level alignment such as kidney position), but instead relies on approximate spatial consistency derived from physical coordinates, with residual discrepancies handled by subsequent registration.

#### 4.1.3. Network Architecture and Implementation Details

The generative network employs a ResNet-based architecture containing 9 residual blocks to facilitate deep feature extraction while preserving spatial resolution. The discriminator utilizes the standard 70 × 70 PatchGAN architecture, which penalizes structural inconsistencies at the scale of image patches rather than the entire image, thereby preserving high-frequency anatomical details. For the patch-wise contrastive learning (PatchNCE), the feature network (netF) was constructed using a 2-layer multi-layer perceptron (MLP) with 256 channels. It sampled 512 patches across nine designated network layers (layers 0 through 8).

The framework was implemented in PyTorch (version 2.3.1) and trained on a single NVIDIA RTX A6000 GPU (48 GB VRAM). Both the generator and the discriminator were optimized using the Adam optimizer ($\beta_{1}=0.5,\beta_{2}=0.999$) with a constant learning rate of $2\times 10^{-4}$. The training process was conducted for 40 epochs with a batch size of 16. The total training time for Phase 1 (pre-training on 156 subjects) was approximately 105 h (379,873 s), while Phase 2 (fine-tuning on 8 low-dose subjects) required approximately 6 h (21,920 s) using the specified hardware configuration.

The comprehensive objective function was balanced using empirical weights carefully tuned for medical image translation. The specific loss weights were assigned as follows: adversarial loss $\lambda_{adv}=1.0$, multi-scale pyramid L1 loss $\lambda_{pyr}=15.0$ (applied across four spatial resolution scales: L1, L2, L3, L4), hard-negative decoupled contrastive loss $\lambda_{HDCE}=0.2$, semantic-relation consistency loss $\lambda_{SRC}=0.1$, and edge-preserving loss $\lambda_{edge}=2.0$. A comprehensive summary of all hyperparameters, including network optimization settings and empirical loss weights, is provided in Table 1 to facilitate reproducibility.

### 4.2. Evaluation Metrics

Enhancement performance was evaluated from three perspectives: (i) reference-anchored fidelity, (ii) perceptual and distributional quality, and (iii) region-level contrast analysis with reliability validation. Test data were organized at the slice level for each subject. All evaluations were performed in grayscale. For FID and LPIPS, grayscale images were replicated across three channels because these metrics are defined for RGB inputs. Results are reported as mean ± standard deviation across all slices in the test set.

CNR (Equation (7)) was used to quantify contrast separability between anatomical structures and a reference region. Here, anatomical structures include the caudal vena cava (CVC), portal vein (PV), and hepatic vein (HV).

(4) $$PSNR=20\log_{10}\left(\frac{I_{\max}}{\sqrt{MSE}}\right),$$

(5) $$MSE=\frac{1}{HW}\sum_{i}{\left(x_{i}-{\hat{x}}_{i}\right)}^{2},$$

where $I_{\max}$ denotes the maximum possible pixel intensity (255 for 8-bit images); $x_{i}$ and ${\hat{x}}_{i}$ represent the pixel intensities of the reference and generated images at index *i*, respectively; and *H* and *W* denote the height and width of the image.

**Reference-anchored fidelity:** PSNR (Equation (4)) and MSE (Equation (5)) quantify reconstruction consistency [30]. The variables $x_{i}$ and ${\hat{x}}_{i}$ denote the pixel intensities of the reference and generated images at index *i*, respectively, while *H* and *W* represent the spatial dimensions of the image, and $I_{\max}$ denotes the maximum possible pixel value (e.g., 255 for 8-bit images). MS-SSIM evaluates structural similarity while accounting for human visual characteristics [31]. Because these metrics were computed against RPR rather than real Post100 images, they are interpreted as reference-anchored consistency measures rather than absolute reconstruction accuracy.

(6) $$FID={∥\mu_{r}-\mu_{g}∥}_{2}^{2}+Tr\left(\Sigma_{r}+\Sigma_{g}-2{\left(\Sigma_{r}\Sigma_{g}\right)}^{\frac{1}{2}}\right),$$

where $\mu_{r}$ and $\mu_{g}$ denote the mean feature vectors of real and generated image distributions, $\Sigma_{r}$ and $\Sigma_{g}$ denote the corresponding covariance matrices, and Tr(·) represents the matrix trace operator.

**Perceptual and distributional quality:** FID (Equation (6)) measures the distance between reference and generated image distributions in Inception-V3 feature space [32], where $\mu_{r}$ and $\mu_{g}$ denote the mean feature vectors, and $\Sigma_{r}$ and $\Sigma_{g}$ denote the corresponding covariance matrices of the reference (*r*) and generated (*g*) distributions, respectively. LPIPS quantifies patch-wise perceptual similarity using an AlexNet backbone [33]. These metrics complement reference-anchored fidelity by capturing global distributional realism and perceptual similarity.

(7) $$CNR=\frac{|\mu_{\mathrm{sig}}-\mu_{\mathrm{ref}}|}{\sigma_{\mathrm{ref}}},$$

where $\mu_{\mathrm{sig}}$ and $\mu_{\mathrm{ref}}$ denote the mean intensities of the signal and reference regions, respectively, and $\sigma_{\mathrm{ref}}$ denotes the standard deviation of the reference region.

**Region-level contrast analysis:** CNR (Equation (7)) was used to quantify contrast separability between anatomical structures and a reference region, where $\mu_{\mathrm{sig}}$ and $\mu_{\mathrm{ref}}$ denote the mean intensities of the target anatomical signal and the reference tissue, respectively, and $\sigma_{\mathrm{ref}}$ denotes the standard deviation of the reference tissue. ROIs were extracted as polygons using LabelMe, and the epaxial muscle above the transverse process served as the reference tissue. Because CNR can be artificially increased by variance suppression or over-smoothing, CNR results were interpreted together with distribution similarity and vessel profile consistency analyses.

(8) $$\mathrm{KLD}\left(P||Q\right)=\sum_{i}P\left(x_{i}\right)\log\frac{P(x_{i})}{Q(x_{i})},$$

(9) $$W\left(P,Q\right)=\underset{\gamma \in \Gamma (P,Q)}{\inf}\int ||x-y||\;d\gamma \left(x,y\right),$$

where *P* and *Q* denote the intensity distributions of the generated and reference images, respectively; $x_{i}$ represents sampled intensity values; Γ(P,Q) denotes the set of all possible transport plans between *P* and *Q*; and γ(x,y) represents a specific transport plan.

**Distribution similarity:** Intensity distribution matching between generated ROIs and RPR ROIs was assessed using Kullback–Leibler divergence (KLD) and Wasserstein distance. In Equations (8) and (9), *P* and *Q* denote the intensity distributions of generated and RPR ROIs, respectively, and $x_{i}$ represents sampled intensity values. Additionally, Γ(P,Q) denotes the set of all joint distributions with marginals *P* and *Q*, and γ(x,y) represents a specific transport plan between the distributions. Lower values indicate better agreement with the reference anchor.

(10) $$\rho =\frac{\mathrm{Cov}(I_{\mathrm{gen}},I_{\mathrm{RPR}})}{\sigma_{I_{\mathrm{gen}}}\sigma_{I_{\mathrm{RPR}}}},$$

where Cov(·) denotes the covariance operator; $I_{\mathrm{gen}}$ and $I_{\mathrm{RPR}}$ represent the intensity profiles of the generated and reference (RPR) images, respectively; and σ denotes the standard deviation.

**Vessel profile consistency:** Cross-sectional vessel intensity profile agreement with RPR was assessed by Pearson correlation (Equation (10)), where $I_{\mathrm{gen}}$ and $I_{\mathrm{RPR}}$ denote intensity profiles extracted from generated and RPR images, respectively. Higher correlation indicates better preservation of vascular intensity patterns.

All evaluation metrics (PSNR, MSE, MS-SSIM, FID, LPIPS, KLD, Wasserstein distance, and profile correlation) were computed on 8-bit PNG images to ensure reproducibility and consistency with common medical image synthesis benchmarks. Statistical significance of the performance differences between the proposed method and the baseline models was evaluated. Because the slice-wise evaluation results were aggregated as summary statistics across 1171 test slices, an independent two-sample *t*-test with unequal variances (Welch’s *t*-test) was employed. A *p*-value of less than 0.05 was considered statistically significant.

#### Baselines

Four representative enhancement and translation methods were retrained and evaluated under identical protocols. All models used a 256×256 input size, identical preprocessing, and the same training and evaluation splits.

**Pix2Pix:** a conditional GAN for paired image translation trained with L1 and adversarial losses [21,34]. In this study, pseudo-pairs generated by hybrid registration were used as supervisory signals, representing a pseudo-supervised baseline under weak pairing.
**CycleGAN:** an unpaired translation framework using cycle-consistency and identity losses [35]. Pre-contrast and post-contrast domains were separated to preserve fully unpaired learning without registration-based pairing.
**NCE (Neural Contrast Enhancement):** a two-stage method that generates registered synthetic NECT–CECT pairs and enables reconstruction-based learning [20]. We followed the original loss configuration and learning strategy while adjusting resolution, batch size, and schedule to match our protocol.
**ADN (Artifact Disentanglement Network):** a framework originally proposed for artifact disentanglement [36,37] and used here as a generic unsupervised translation baseline. Its architecture was preserved while the objective was adapted to reconstruction and adversarial losses.

### 4.3. Comparative Results

Table 2 summarizes the comparative enhancement results in terms of reference-anchored fidelity and perceptual quality. All methods were evaluated against RPR, which serves as a registration-based anchor rather than ground truth. Accordingly, these metrics were interpreted together with region-level and distributional analyses. Relative to the low-enhanced Post40 input, the proposed method improved PSNR by 2.7 dB, increasing from 21.4 dB to 24.1 dB while maintaining structural plausibility.

**Figure 3:**
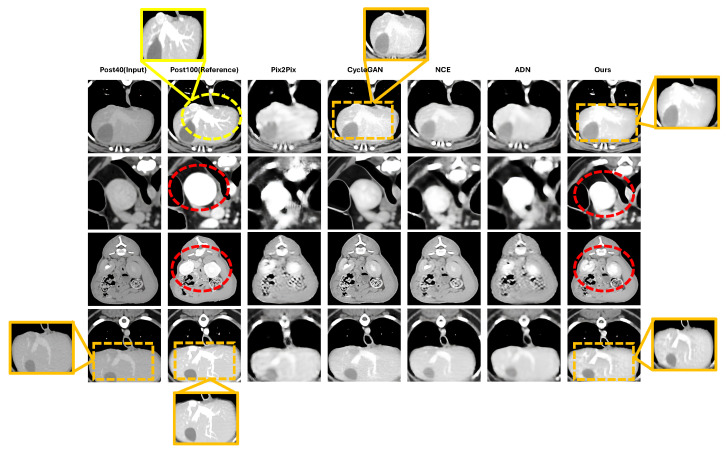
Qualitative comparison of dose-up translation results from Post40 inputs. Results for each method (Pix2Pix, CycleGAN, NCE, ADN, and the proposed method) are shown together with the registration-based pseudo-reference (RPR, Post100). Yellow dashed circles indicate regions where enhancement remains challenging for all methods due to limited contrast and complex anatomical structures. Red dashed circles highlight regions where the proposed method provides clearer structural delineation, including improved vessel depiction and sharper organ boundaries. The orange boxes indicate magnified regions of interest (ROIs) for detailed comparison.

Qualitative comparisons in Figure 3 support these quantitative findings. The proposed method shows clearer vascular continuity, sharper vessel boundaries, and more homogeneous parenchymal enhancement than the baseline methods, particularly in the highlighted vascular regions. By contrast, several baselines exhibit blurring, patch-level inconsistency, or incomplete enhancement in regions affected by weak spatial correspondence.

In regions with very limited enhancement in the Post40 input (yellow dashed circles), all methods showed restricted recovery. This finding reflects a practical limitation of Post40-to-Post100 enhancement under weak supervision, where source-domain contrast information may be insufficient to fully reconstruct high-dose appearance.

The proposed method achieved the lowest FID (38.584), indicating superior distributional consistency relative to both the low-enhanced input and competing methods. Although the input baseline showed competitive structural metrics, its higher FID suggests that geometric alignment alone is insufficient for recovering diagnostically useful enhancement patterns. The proposed framework successfully addresses this limitation through reference-guided enhancement and structure-aware regularization.

Furthermore, while our method demonstrated statistically significant improvements in structural metrics over the best-performing baseline (NCE), the perceptual similarity metric (LPIPS: 0.065) showed no statistically significant difference (p>0.05). This aligns with the theoretical nature of LPIPS, which primarily evaluates global perceptual realism rather than strict voxel-wise alignment. Because the NCE baseline utilizes adversarial training, it already achieves strong perceptual quality, albeit with localized spatial misalignments. Our framework effectively corrects these structural discrepancies—evidenced by significant gains in PSNR, MS-SSIM, and MSE—while maintaining the same high level of perceptual realism. Thus, the comparable LPIPS scores confirm that our method enhances anatomical fidelity without compromising the visual naturalness of the generated enhancement patterns.

### 4.4. Region-Level Contrast Analysis

CNR reflects region-level contrast separability rather than voxel-wise fidelity. Because RPR serves as a registration-based anchor rather than ground truth, CNR values exceeding those of RPR do not necessarily indicate hallucinated enhancement. They may instead reflect improved contrast differentiation achieved through noise suppression while maintaining anatomical structure.

CNR was measured across 4 test subjects with 3–4 ROIs per organ. ROI-level values were aggregated across slices and subjects. To reduce bias caused by model-dependent variance changes, the reference region (epaxial muscle) was extracted from input images rather than generated outputs.

**Figure 4:**
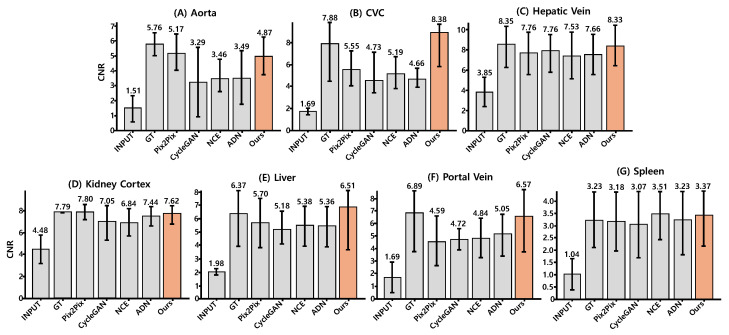
Region-wise variability of contrast-to-noise ratio (CNR) across seven anatomical regions. Bars represent mean CNR aggregated across slices and test subjects, and error bars indicate standard deviation. Higher CNR reflects improved contrast separability between anatomical structures and reference tissue; consistent with Table 3, these values are interpreted as relative enhancement rather than absolute fidelity due to pseudo-reference anchoring. Orange bars correspond to the proposed method.

As shown in Figure 4 and Table 3, the proposed method achieved an average CNR of 6.52 across seven anatomical regions, exceeding Pix2Pix (5.68) and the other baselines, and substantial improvements over the low-enhanced Post40 input (2.32). The proposed method yielded the highest CNR in four of seven regions: Caudal Vena Cava (CVC), hepatic vein, liver, and portal vein. In the remaining regions, performance remained close to the best baseline values.

In vascular structures, the proposed method approached RPR in aorta (4.87 vs. 5.76), hepatic vein (8.33 vs. 8.35), and portal vein (6.57 vs. 6.89), and slightly exceeded RPR in CVC (8.38 vs. 7.88). This behavior is consistent with the definition of CNR, in which both signal separability and reference-region noise contribute to the final value. Because RPR may contain residual noise introduced by deformable registration, modest CNR gains beyond RPR can arise from structure-preserving enhancement combined with reduced reference-region variance. This interpretation was further examined using distribution similarity and vessel profile consistency analyses.

### 4.5. Validation of Contrast Reliability

To assess whether the observed CNR improvements reflected plausible enhancement rather than numerical artifacts, distribution similarity and vessel profile consistency were evaluated (Table 4). The proposed method achieved the lowest divergence from RPR, with a KLD of 3.25 and a Wasserstein distance of 10.83, indicating improved agreement in ROI intensity distributions.

The proposed method also maintained the vessel profile correlation (0.35), suggesting preservation of anatomically consistent vascular intensity patterns. Together, these results support the interpretation that the observed contrast gains arise from enhancement consistent with the RPR anchor rather than from excessive smoothing or variance collapse, which would typically degrade distributional fidelity and profile agreement.

### 4.6. Ablation Studies

#### 4.6.1. Effect of Hybrid Registration

We first examined the contribution of registration quality to enhancement performance. The registration-only baseline (Input in Table 2) achieved 21.4 dB PSNR against RPR, whereas the complete framework reached 24.1 dB, indicating that enhancement contributes beyond alignment alone.

Table 5 further isolates the contribution of hybrid registration. ANTs-SyN alone achieved 22.62 dB PSNR and 469.2 MSE, whereas the addition of DINO-Reg improved performance to 23.77 dB PSNR and 357.6 MSE. This corresponds to a 1.15 dB PSNR gain and a 23.8% reduction in MSE. To isolate the registration effect, edge loss was omitted in this experiment.

**Figure 5:**
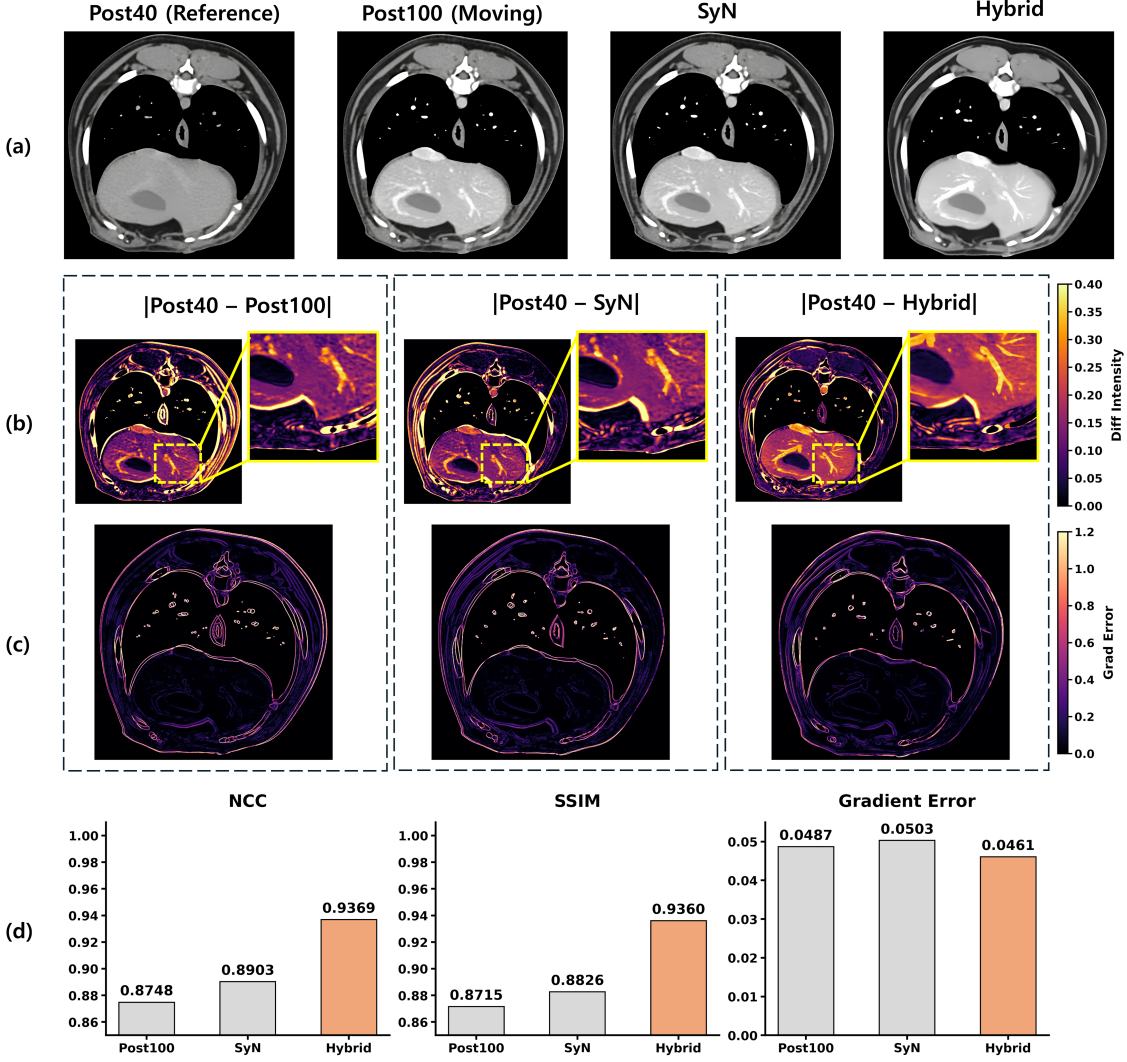
Qualitative and quantitative evaluation of spatial alignment and structural preservation. (**a**) Axial CT slices of the reference (Post40), moving image (Post100), and aligned results using global deformable registration (SyN) and the proposed hybrid method (Hybrid). (**b**) Absolute difference maps with respect to Post40, where Post40–Post100 serves as a baseline for initial misalignment. ROIs highlight residual local discrepancies. (**c**) Gradient difference maps illustrating boundary-level mismatches. (**d**) Quantitative comparison using NCC, SSIM, and Gradient Error, computed with respect to Post40. The hybrid method achieves improved alignment over both Post100 and SyN. Bold values indicate the best performance for each metric.

Qualitative comparisons in Figure 5 illustrate the same tendency. The SyN-only strategy achieves coarse anatomical alignment but leaves residual local inaccuracies, particularly along vascular and soft-tissue boundaries. The proposed hybrid strategy improves local correspondence and reduces misalignment artifacts in these anatomically complex regions.

As observed in Figure 5, the slice-wise application of ANTs-SyN can occasionally yield images that appear globally identical to the moving Post100 target. However, this macroscopic similarity often masks subtle, local misalignments along complex vascular and soft-tissue boundaries. When these SyN-only images are used as supervisory anchors, such residual discrepancies degrade training stability and cause boundary blurring in the final generative output. The proposed hybrid strategy explicitly mitigates this limitation; the subsequent DINO-Reg refinement corrects these local feature-level mismatches, providing a highly reliable pseudo-reference that is essential for preserving structural fidelity during training.

#### 4.6.2. Effect of Loss Configuration

We further examined the contribution of reconstruction loss configurations across the two training stages (Table 6). In Phase 1 (Pre → Post100), adding edge-preserving loss improved PSNR under both single-scale and multi-scale reconstruction settings, indicating the importance of boundary preservation when bridging larger domain differences.

In Phase 2 (Post40 → Post100), the Edge + Pyramid L1 configuration achieved the best performance, reaching 24.13 dB PSNR and 852.8 MSE. Compared with L1 alone, this corresponds to a 1.39 dB gain in PSNR and a reduction of 128.1 in MSE. Although Edge + Pyramid L1 showed a slight decrease relative to Edge + L1 in Phase 1, it produced the largest gain during low-dose adaptation, suggesting that multi-scale consistency is particularly beneficial when residual differences are localized and intensity-driven.

#### 4.6.3. Incremental Pipeline-Wise Ablation Study

To rigorously isolate the contributions of the key proposed modules and demonstrate their incremental improvements, we conducted a pipeline-wise ablation study. The evaluation progressively integrated the hybrid registration, structure-aware regularization (multi-scale pyramid L1 and edge-preserving losses), and the two-stage knowledge transfer strategy. The baseline model was defined as a single-stage translation network utilizing only global deformable alignment (ANTs-SyN) and a standard L1 reconstruction loss without pre-training.

As summarized in Table 7, the baseline configuration exhibited limited structural fidelity (PSNR: 21.75 dB) and struggled with distributional realism (FID: 75.11).

**Impact of Hybrid Registration:** Introducing the DINO-Reg feature-level refinement (+Hybrid Regist.) led to a substantial reduction in spatial discrepancies. This single addition provided the most significant performance leap, improving PSNR by 1.42 dB (from 21.75 to 23.17) and decreasing MSE from 1319.1 to 931.0. The dramatic drop in FID (from 75.11 to 47.49) underscores the necessity of precise local alignment for generating realistic enhancement patterns.

**Impact of Structure-Aware Regularization:** Building upon the hybrid registration, the integration of edge-aware and multi-scale pyramid L1 losses (+ Structure Loss) further refined the anatomical boundaries. This configuration improved the MS-SSIM from 0.906 to 0.912 and raised the PSNR to 23.90 dB, demonstrating that structural constraints are essential for preventing the blurring of delicate vessels and organ interfaces during translation.

**Impact of Two-Stage Knowledge Transfer (Ours):** Finally, transitioning from a single-stage approach to the proposed two-stage knowledge transfer framework yielded the ultimate performance. By leveraging the robust anatomical priors established during the pre-training phase (*n* = 156), the final model effectively overcame the data scarcity of the low-dose target domain. This resulted in the best overall metrics, reaching a PSNR of 24.10 dB, an MSE of 852.8, and the lowest FID of 38.60, confirming that the modules act synergistically to achieve anatomically plausible dose-up translation.

Notably, all structural metrics (PSNR, MS-SSIM, MSE) demonstrated statistically significant improvements over the baseline. Interestingly, the perceptual similarity metric (LPIPS) showed no statistically significant differences across the incremental additions, suggesting that while structural and distributional fidelity improved dramatically, the base perceptual realism was maintained throughout the pipeline. This confirms that the proposed modules act synergistically to achieve anatomically plausible dose-up translation without introducing perceptible artifacts.

## 5. Discussion

This study shows that Post40-to-Post100 CT enhancement remains feasible even when spatial correspondence between acquisitions is incomplete. Under such conditions, conventional pixel-supervised learning becomes unreliable because anatomical misalignment distorts local intensity relationships and compromises structure preservation. The present results indicate that meaningful contrast improvement can still be achieved by combining weakly aligned reference construction, structure-preserving enhancement, and staged knowledge transfer.

Viewed from this perspective, the dose-up translation of low-enhanced scans should not be framed as a conventional reconstruction task that assumes strict voxel-level correspondence. Instead, it is more appropriately interpreted as a domain adaptation problem performed under incomplete structural alignment and uneven contrast attenuation patterns. In this setting, preserving anatomical plausibility and robustness to acquisition variability becomes more critical than enforcing exact voxel-wise fidelity.

From an imaging perspective, this formulation is important because it reflects the conditions of routine veterinary CT more closely than strictly paired reconstruction settings. In practice, repeated acquisitions are affected by respiratory motion, postural variability, and uneven contrast attenuation patterns, all of which reduce the validity of voxel-wise supervision. By operating with reference-aligned anchors rather than exact targets, the proposed framework shifts the emphasis from idealized reconstruction toward practical enhancement under acquisition variability. Practically, this enables retrospective utilization of routinely acquired low-dose scans without requiring protocol-standardized repeat acquisitions or labor-intensive manual pairing. Such compatibility with existing acquisition workflows may expand the usability of clinical imaging archives for contrast enhancement and downstream diagnostic applications.

A central component of this formulation is the registration-based pseudo-reference (RPR), which serves as an anchor for enhancement rather than as a true ground-truth target. For this reason, improvements in region-level CNR should be interpreted primarily as gains in contrast separability rather than as direct evidence of voxel-wise fidelity. Under this interpretation, CNR values that approach or slightly exceed those of RPR do not necessarily indicate artificial signal amplification. Instead, they may reflect improved differentiation between enhanced structures and reference tissue, particularly when noise in the reference region is reduced while anatomical boundaries are preserved.

The complementary validation analyses support this interpretation. The proposed method showed lower Kullback–Leibler divergence and Wasserstein distance than the comparison methods, indicating improved agreement between generated and reference intensity distributions within anatomically defined ROIs. Vessel profile correlation was also maintained, suggesting that local vascular intensity patterns remained structurally plausible. Taken together, these findings support the view that the observed contrast gains arise from anatomically consistent enhancement rather than from over-smoothing or spurious intensity amplification.

The results also highlight a practical distinction between alignment and enhancement. Although deformable registration improved coarse correspondence, registration alone was insufficient to recover diagnostically useful enhancement patterns from low-dose scans. This was evident in the relatively high FID of the low-enhanced input despite competitive structural metrics. In other words, anatomical alignment can reduce geometric discrepancy, but it cannot compensate for missing or weak contrast information. The proposed framework addresses this limitation by combining reference-guided enhancement with structural constraints that preserve vessels and organ boundaries.

Several limitations should be considered. First, the dataset remains limited in size, with 168 subjects in total and a small number of low-dose cases available for adaptation and evaluation. In particular, the number of evaluation subjects is small (*n* = 4), limiting subject-level statistical power; therefore, the observed performance differences should be interpreted as preliminary trends rather than statistically confirmed effects. Although the staged transfer strategy partially alleviates this limitation by leveraging abundant Pre-to-Post100 data, broader validation is still required to assess generalizability across breeds, body sizes, and imaging settings. Consequently, further validation on larger subject-level cohorts is necessary to establish generalizability.

The choice of comparison methods should also be interpreted in light of the imaging conditions addressed in this study. The selected baselines represent paired, unpaired, synthetic-pairing, and unsupervised enhancement settings that remain relevant under limited or imperfect correspondence. In settings characterized by respiratory motion, protocol variability, and inconsistent contrast timing, methods that rely on precise structural conditioning implicitly assume anatomical stability that is rarely achieved in routine practice. Under such violations, strict pixel-aligned supervision may amplify misregistration artifacts or encourage anatomically inconsistent synthesis, limiting their reliability in weakly aligned clinical datasets. For weakly aligned low-dose enhancement, preserving anatomical plausibility under acquisition inconsistency is more critical than maximizing synthesis fidelity under idealized conditions.

Future work should extend this framework in several directions. First, multi-institutional evaluation across species and pathological conditions is needed to assess robustness under broader clinical variability. Second, uncertainty-aware enhancement strategies may help identify regions where source-domain contrast information is insufficient for reliable recovery. Third, although we incorporated region-level CNR, distributional distances, and vessel profile correlations as quantitative clinical proxies to overcome the limitations of traditional pixel-wise metrics, these ultimately remain surrogate measures. Consequently, prospective reader studies with expert radiological assessment are planned as an exploratory next step to determine whether these proxy-driven improvements directly translate into enhanced diagnostic efficacy within real-world clinical workflows.

Beyond performance improvement, this study introduces a practical conceptual shift: dose-up translation under imperfect acquisition conditions should prioritize anatomical plausibility and robustness over strict reconstruction fidelity. This perspective may guide the development of future enhancement frameworks designed for real-world imaging environments where ideal supervision and precise spatial correspondence are unattainable.

Overall, the present findings suggest that low-dose contrast-enhanced CT can be improved using routinely acquired imaging data even when strict spatial correspondence is unavailable. This supports a more practical direction for CT enhancement research, one that emphasizes anatomical plausibility, robustness to acquisition variability, and compatibility with real-world imaging conditions.

## 6. Conclusions

This study presented a weakly aligned enhancement framework for Post40-to-Post100 CT acquired under spatial misalignment and limited paired data. By integrating reference-guided alignment, staged knowledge transfer, and structure-preserving enhancement, the proposed approach achieved anatomically consistent contrast improvement across vascular and parenchymal regions despite incomplete geometric correspondence.

These findings suggest that low-dose contrast enhancement under routine clinical acquisition should not be regarded as a conventional reconstruction task requiring strict voxel-level correspondence. Instead, it can be addressed as a practically constrained domain adaptation problem in which anatomical plausibility and robustness to acquisition variability are prioritized over pixel-wise fidelity.

Because the registration-based pseudo-reference (RPR) serves as an enhancement anchor rather than a true ground-truth target, performance was evaluated using complementary structural and distributional criteria rather than relying solely on reconstruction metrics. Region-level contrast analysis, intensity distribution similarity, and vessel profile consistency indicate that the observed improvements correspond to anatomically plausible enhancement rather than numerical artifacts.

Several limitations remain. The current framework does not explicitly model volumetric consistency and shows reduced effectiveness in extremely low-contrast regions where source-domain information is insufficient. Future work will investigate volumetric consistency modeling, incorporation of multi-phase contrast information, and prospective clinical validation in pathological cases. Future work will also focus on optimizing the computational efficiency of the framework. While the current 2D ResNet-based design offers a practical balance between depth and memory usage, we aim to investigate lightweight architectures through model pruning and knowledge distillation. Such optimizations will be essential for deploying the dose-up translation framework on resource-constrained hardware for real-time clinical applications.

## Figures and Tables

**Table 1:** Summary of hyperparameters used for training the proposed framework.

Parameter	Value	Description
Optimizer	Adam	$\beta_{1}=0.5,\beta_{2}=0.999$
Learning Rate	$2\times 10^{-4}$	Constant
Batch Size	16	-
Epochs	40	Phase 1 & Phase 2
Input Res.	256×256	Resized for GPU
$\lambda_{adv}$	1.0	PatchGAN weight
$\lambda_{pyr}$	15.0	Multi-scale pyramid L1 consistency
$\lambda_{edge}$	2.0	Gradient consistency
$\lambda_{HDCE}$	0.2	Hard-negative decoupled
$\lambda_{SRC}$	0.1	Semantic consistency
PatchNCE	Layers 0–8	512 patches, 2-layer MLP

**Table 2:** Quantitative comparison of dose-up translation performance for low-dose CT (Post40). All methods were evaluated against registration-based pseudo-reference images (RPRs, derived from Post100). Input denotes low-enhanced Post40 images. Values are reported as mean (standard deviation) across test slices, except for FID, which is computed at the dataset level and reported as a single aggregate value. Bold values indicate the most favorable results for each metric. Statistical significance for slice-level metrics (PSNR, MS-SSIM, MSE, and LPIPS) was evaluated using Welch’s *t*-test, with * indicating statistical significance (p<0.05) for our proposed method compared to the best-performing baseline (NCE).

Method	PSNR ↑	MS-SSIM ↑	MSE ↓	FID ↓	LPIPS ↓
Input	21.4 (2.9)	0.898 (0.043)	1389.3 (778.9)	69.1	0.096 (0.033)
Pix2Pix [17]	22.3 (2.7)	0.891 (0.041)	1092.8 (552.7)	81.2	0.091 (0.022)
CycleGAN [35]	22.9 (3.4)	0.902 (0.047)	971.2 (530.5)	52.1	0.073 (0.035)
NCE [20]	23.7 (3.2)	0.910 (0.045)	861.8 (557.6)	48.3	**0.065** (0.022)
ADN [36,37]	22.9 (3.2)	0.899 (0.045)	999.7 (568.9)	68.7	0.085 (0.024)
**Ours**	**24.1** * **(3.8)**	**0.914** * **(0.052)**	**852.8** * **(618.0)**	**38.6**	**0.065** **(0.026)**

**Table 3:** Mean region-level contrast-to-noise ratio (CNR) across seven anatomical regions (Aorta, Caudal Vena Cava (CVC), Hepatic Vein, Portal Vein, Spleen, Liver, and Kidney Cortex). CNR was computed using the epaxial muscle as a consistent reference region to enable standardized comparison across slices, subjects, and methods. Values represent mean CNR aggregated across slices and test subjects. Higher CNR indicates improved contrast separability between anatomical structures and reference tissue; however, because evaluation is anchored to registration-based pseudo-references (RPRs) rather than true ground truth, CNR values are interpreted as relative dose-up translation rather than absolute fidelity. The RPR row represents reference-aligned images provided for comparison. Bold values indicate the highest CNR for each anatomical region.

Method	Aorta	CVC	Hepatic Vein	Kidney Cortex	Liver	Portal Vein	Spleen
INPUT	1.51	1.69	3.85	4.48	1.98	1.69	1.04
RPR	5.76	7.88	8.35	7.79	6.37	6.89	3.23
Pix2Pix [17]	**5.17**	5.55	7.76	**7.80**	5.70	4.59	3.18
CycleGAN [35]	3.29	4.73	7.76	7.05	5.18	4.72	3.07
NCE [20]	3.46	5.19	7.53	6.84	5.38	4.84	**3.51**
ADN [36,37]	3.49	4.66	7.66	7.44	5.36	5.05	3.23
**Ours**	4.87	**8.38**	**8.33**	7.62	**6.51**	**6.57**	3.37

**Table 4:** Distribution-and profile-based validation metrics supporting CNR-based contrast analysis. Kullback–Leibler divergence (KLD) and Wasserstein distance quantify intensity distribution similarity between enhanced images and the registration-based pseudo-reference (RPR) across all analyzed vessel and organ regions of interest. Prof. Corr. denotes Pearson correlation of cross-sectional vessel intensity profiles between enhanced and RPR images. Values represent mean (standard deviation) aggregated across all regions and test subjects. Lower KLD and Wasserstein distance and higher Prof. Corr. indicate closer agreement with the RPR reference. Bold values indicate the best performance for each metric (lower is better for KLD and Wasserstein distance, higher is better for Prof. Corr.).

Method	KLD ↓	Wass. ↓	Prof. Corr. ↑
Pix2Pix [17]	5.86 (4.03)	19.27 (11.36)	0.37 (0.40)
CycleGAN [35]	5.39 (4.78)	17.76 (11.71)	0.31 (0.36)
NCE [20]	6.60 (4.66)	23.28 (12.81)	0.26 (0.42)
ADN [36,37]	6.19 (4.83)	21.59 (12.68)	0.19 (0.39)
**Ours**	**3.25 (2.97)**	**10.83 (9.48)**	**0.35 (0.39)**

**Table 5:** Ablation study evaluating the impact of the hybrid registration strategy. PSNR and MSE were compared between the base configuration with ANTs-SyN alone and the hybrid configuration with additional DINO-Reg refinement (ANTs-SyN + DINO-Reg), with edge-preserving loss excluded to isolate the effect of registration quality. Values are reported as mean (standard deviation) across test slices. Statistical significance was evaluated at the slice level (*N* = 1171) using Welch’s *t*-test (* indicates *p* < 0.001 compared to the Base with ANTs-SyN). Bold values indicate the best performance for each metric.

Method	PSNR ↑	MSE ↓
Base with ANTs-SyN	22.62 (3.64)	469.2 (302.6)
+ DINO-Reg (Hybrid)	**23.77** * **(3.38)**	**357.6** * **(244.7)**

**Table 6:** Ablation study of reconstruction loss configurations across the two-stage knowledge transfer framework. Phase 1 (Pre → Post100) evaluates loss combinations for learning general contrast characteristics from full-dose data, whereas Phase 2 (Post40 → Post100) examines their effectiveness for adapting to low-dose enhancement under weak alignment. Combinations of L1, edge-preserving loss, and multi-scale (pyramid) consistency were compared. Performance is reported using average PSNR and MSE across test slices. Statistical significance was evaluated at the slice level (*N* = 1171) using Welch’s *t*-test, with an asterisk (*) indicating *p* < 0.05 compared to the Phase 1 L1. Bold values indicate the best performance for each metric.

Method	PSNR ↑	MSE ↓
**Phase 1**		
L1	22.28	1061.5
Edge + L1	23.61 *	884.1 *
Pyramid L1	22.75 *	995.8 *
Edge + Pyramid L1	23.55 *	906.6 *
**Phase 2**		
L1	22.74 *	980.9 *
Edge + L1	23.62 *	888.4 *
Pyramid L1	23.28 *	922.3 *
Edge + Pyramid L1	**24.13** *	**852.8** *

**Table 7:** Incremental pipeline-wise ablation study demonstrating the contribution of each proposed module. The baseline consists of single-stage training with ANTs-SyN global registration and standard L1 loss. Modules were incrementally added to show step-by-step improvements in reference-anchored fidelity and distributional realism. Statistical significance for structural metrics (PSNR, MS-SSIM, MSE) and perceptual metrics (LPIPS) was evaluated at the slice level (*N* = 1171) using Welch’s *t*-test, with an asterisk (*) indicating a statistically significant difference (*p* < 0.001) compared to the Base model. Bold values indicate the best performance for each metric.

Model	Hybrid	Structure	Knowledge	PSNR ↑	MS-SSIM ↑	MSE ↓	FID ↓	LPIPS ↓
	Regist.	Loss	Transfer					
Base				21.75 (3.3)	0.884 (0.058)	1319.1 (838.1)	75.11	0.068 (0.036)
+ Hybrid Regist.	✓			23.17 * (2.9)	0.906 * (0.047)	931.0 * (543.9)	47.49	0.069 (0.021)
+ Structure Loss	✓	✓		23.90 * (3.6)	0.912 * (0.053)	864.2 * (596.3)	44.20	0.066 (0.025)
**+ Knowledge Transfer (Ours)**	✓	✓	✓	**24.10** * **(3.8)**	**0.914** * **(0.052)**	**852.8** * **(618.0)**	**38.60**	**0.065 (0.026)**

## Data Availability

The data used in this study are not publicly available due to privacy and institutional restrictions but may be available from the corresponding authors upon reasonable request.
